# Simplified finite model based evaluation of tissue stress distribution on anesthetic feet of Leprosy patients for 3 dimensional orthosis fabrication

**DOI:** 10.1186/1757-1146-7-S1-A91

**Published:** 2014-04-08

**Authors:** Sathish K Paul, Sudesh Sivarasu

**Affiliations:** 1 VIT University, Vellore - 632014, Tamil Nadu, India; 2 Lecturer & Project leader, Biomechanics, University of Cape Town, Cape Town 7925, South Africa

## Background

The Subtalar joint position during static stance is a crucial determinant of the peak plantar pressures and forms a base for any intervention in foot related problems for leprosy affected patients[1]. Studies have stated that the subtalar joint when in neutral position is more ideal for orthotic fabrication. In this study a hypothesis was formulated and pursued [2,3]. Central to the hypothesis is that the stress will be minimal in the distal joints of the foot when the subtalar joint is neutral at static stance position.

## Results

The Computed Tomography (CT) images of the feet for 5 patients suffering from Hansen’s disease having no muscle weakness and joint restriction were acquired. The gray intensities corresponding to the bones of the foot from the CT images were 3 dimensionally reconstructed. The three dimensional model of the human foot, incorporating the realistic geometry and the material properties of the hard tissues were then analyzed using a finite element solver. Stress distribution on bones of the foot while on static stance with the subtalar joint in neutral position were acquired. The results demonstrate that the weight of the patient and the position of the calcaneum in the static stance position contribute to the high stresses in the foot. The stresses in the bones of the foot are minimal when the subtalar is in neutral position, suggesting that this position is an optimal aim for foot orthotic fabrication.

## Conclusion

The automating process of designing a customized orthosis with the impression got from the 3 dimensionally modeled feet reduced the modeling time considerably. The simple technique used will help in giving comfort and stability to the patient’s feet while walking.
